# Virchow’s Views on Tubercle

**Published:** 1853-03

**Authors:** 


*Virchow's Views on Tubercle.*—Virchow, in late papers on this subject, advances the opinion that tubercle is the result of a metamorphosis of organized elements. The following is the summary of his views.

"1. Tuberculization, the indubitably local process by which the body described by the name tubercle is formed, is not a peculiar specifice exudation, but a peculiar transformation of tissue elements, such as in 1847 I described in regard of cancer under the name of tuberculous metamorphosis.

"2. The tuberculous metamorphosis is therefore co-ordinate with the fatty and the waxy metamorphosis, calcification, and atheromatous degeneration, but in no way co-ordinate with inflammation or serous effusion, and even less so with suppuration or with cancerous formation.

"3. The tuberculous metamorphosis sometimes affects newly-formed pathological tissues, sometimes the primitive, the so-called physiological tissues, and finally, sometimes it affects both old and new simultaneously, and this last is its ordinary and peculiar characteristic. The tuberculous metamorphosis attacks cellular and transitory as well as fibrous and permanent elements.

"4. The tuberculous metamorphosis consists in a cessation of the nutritive and formative processes, in a mortification, death of the elements of the tissue, with subsequent peripheric absorption of the fluid constituents, and drying-up of the parts lying beyond the sphere of nutrition; the death itself of the elements of the tissue is caused by the accumulation of cell-elements, and is immediately determined by compression of the vessels of the part.

"5. These cells may arise from an absolutely new formation, or from an increased formation of the normal elements (epithelia, enchymkömer, &c.,) or, finally, from an endogenous formation (ans . einer endogenen Bildung.)

"6. All these processes presuppose definite disturbances of the local nutrition, especially an altered exudation, and point back, accordingly, either to inflammation itself or to an analogous affection, no matter whether they owe their origin to an irritation produced by local mischief, or to an excitation consecutive to constitutional causes, primary changes of the blood, &c.

"7. There is therefore an inflammatory, cancerous, typhous, glanderous, sarcomatous, &c. tuberculization, which are altogether the same in reference to the essence of the local process, so far as this depends on tissue metamorphosis, but which are more or less different in reference to the essence of the whole process, as well so far as the latter is local (disturbance of nutrition, exudation, &c.,) as also when it is due to general constitutional causes.

"8. Tuberculosis is the whole process of the affection, comprising the conditions of the local disturbances of the process of nutrition with the changes appertaining to it in the exudation, both in regard of the cell-formation and transformation, and finding in tuberculization its constant regular expression. Every tuberculization (tuberculous metamorphosis) does not have its origin in tuberculosis; tuberculosis can be present, as far as its early stages (exudation, cell-formation) are concerned, and yet there may be no tubercle. We shall therefore call that diseased process tuberculosis, which in its ordinary course always leads to tuberculization; while we shall ascribe cancer and sarcoma, which accidentally tubercularize, to an altogether different process, and shall never give the name of tubercle to a thickened abscess, pus become cheesy, or pus concret.

"9. Scrofulosis is the constitutional affection which, after glanders and typhus, the most frequently produces tuberculosis—i. e., the local disease with the regular termination in tuberculization. But all its products are not tuberculous; tuberculosis is rather co-ordinate with a succession of other local processes.

"10. As tubercle is everywhere formed by the accumulation in the tissues of cells of the most varied kinds, these cells in the majority of cases breaking up, it has no peculiar characteristic elements. The shrivelled nuclei arising from the remains of the cells exhibit the greatest degree of constancy in their outward characters, and therefore we can retain for them the name of tubercle-corpuscles."—*Brit. and For. Med. Rev.*

